# Supplementary figures and images for: The evolutionarily conserved PhLP3 is essential for sperm development in Drosophila melanogaster
Source: PLoS One. 2024 Oct 31;19(10):e0306676. doi: 10.1371/journal.pone.0306676 (PMC11527243; doi:10.1371/journal.pone.0306676)

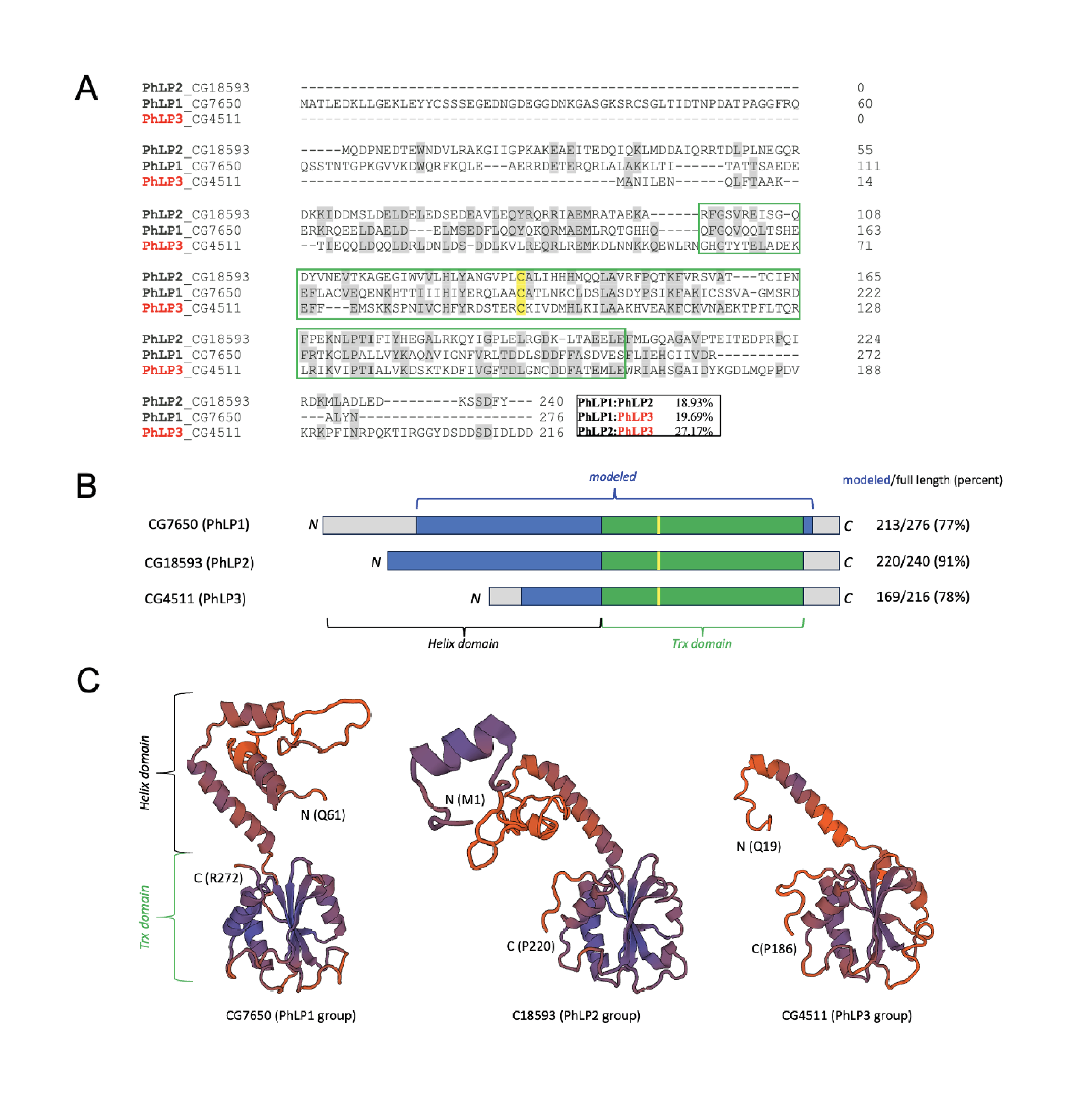

Supplement: S1 Fig — (A) Clustal Omega alignment of the three predicted PhLPs [15, 20]. The conserved residues are indicated with gray boxes. The green boxes indicated the position of the thioredoxin domain with the putative redox-active cysteine highlighted in yellow. The percent identity between each sequence is shown in the box. (B) Comparison of the three PhLPs. Length and characteristic organization of the proteins are indicated. The blue bracket indicates the portion of each protein that was modeled. (C) Hypothetical models of the three predicted PhLP proteins in D. melanogaster. The models were generated using SWISS-MODEL template PDB 7nvm: entity 11, human PhLP2A The structures are colored by confidence level from high (blue) to low (red) [17, 34]. (TIF) [file pone.0306676.s001.tif]

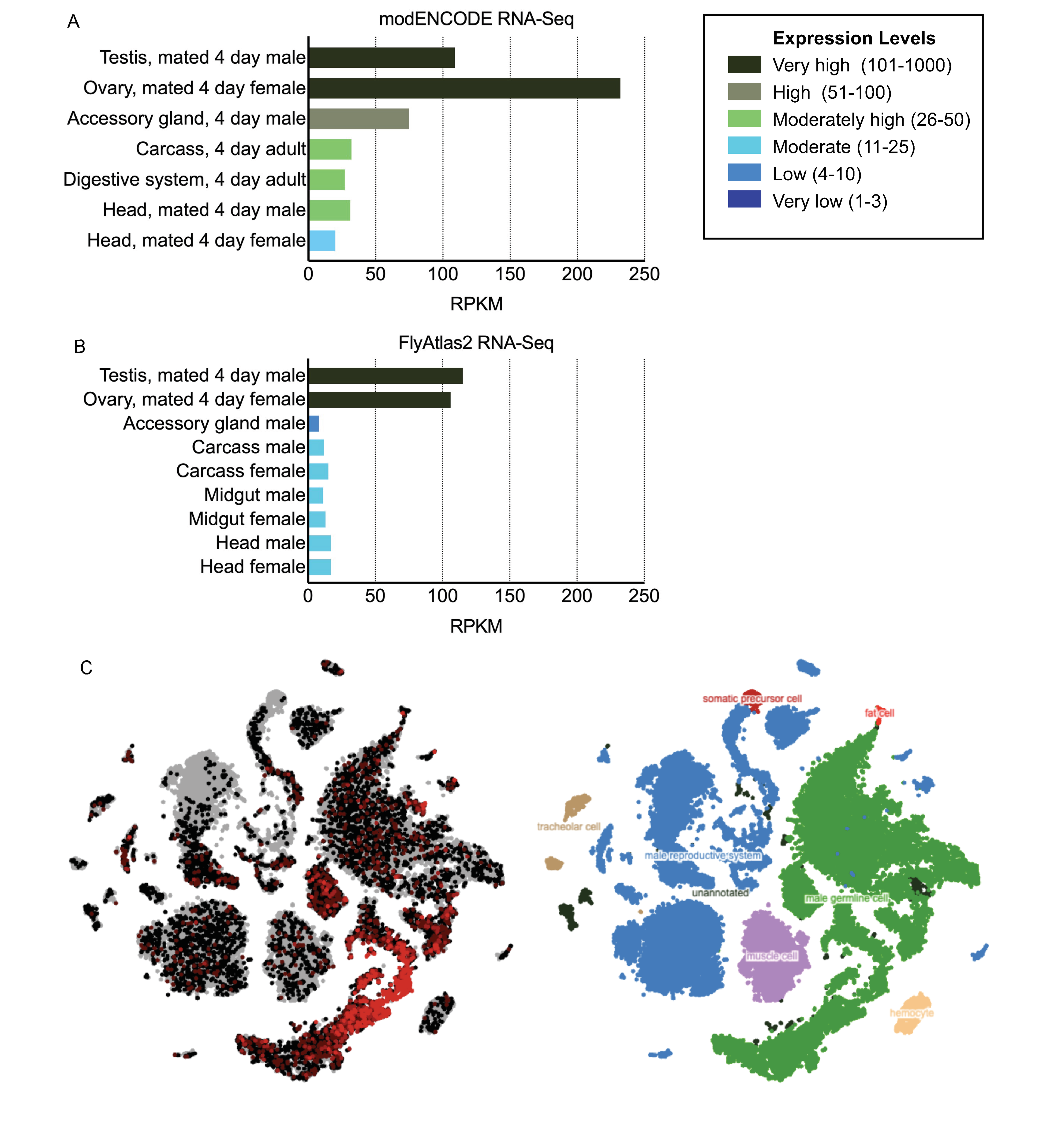

Supplement: S2 Fig — (A) RNA-Seq expression data from select adult dissected tissues from the modENCODE project. Values listed are Reads per kilobase of transcript per Million reads (RPKM) [21]. (B) RNA-Seq expression data from select adult dissected tissues from the FlyAtlas2 project. Values listed are Reads per kilobase of transcript per Million reads (RPKM) [22]. We used FlyBase (FB2023_06, released December 12, 2023) to obtain the data presented in this figure. (C) Single-cell RNA-Seq expression data in the adult testis from the Fly Cell Atlas project is displayed on Uniform Manifold Approximation and Project (UMAP) graph [23]. In the left panel, shades of red indicate expression levels with brightest red being the highest expression and black being low expression. The UMAP in the right panel illustrates the broadly classified different cell clusters in the testis. PhLP3 is most highly expressed in the male germline cells. UMAPs were generated in SCope [81]. (TIF) [file pone.0306676.s002.tif]

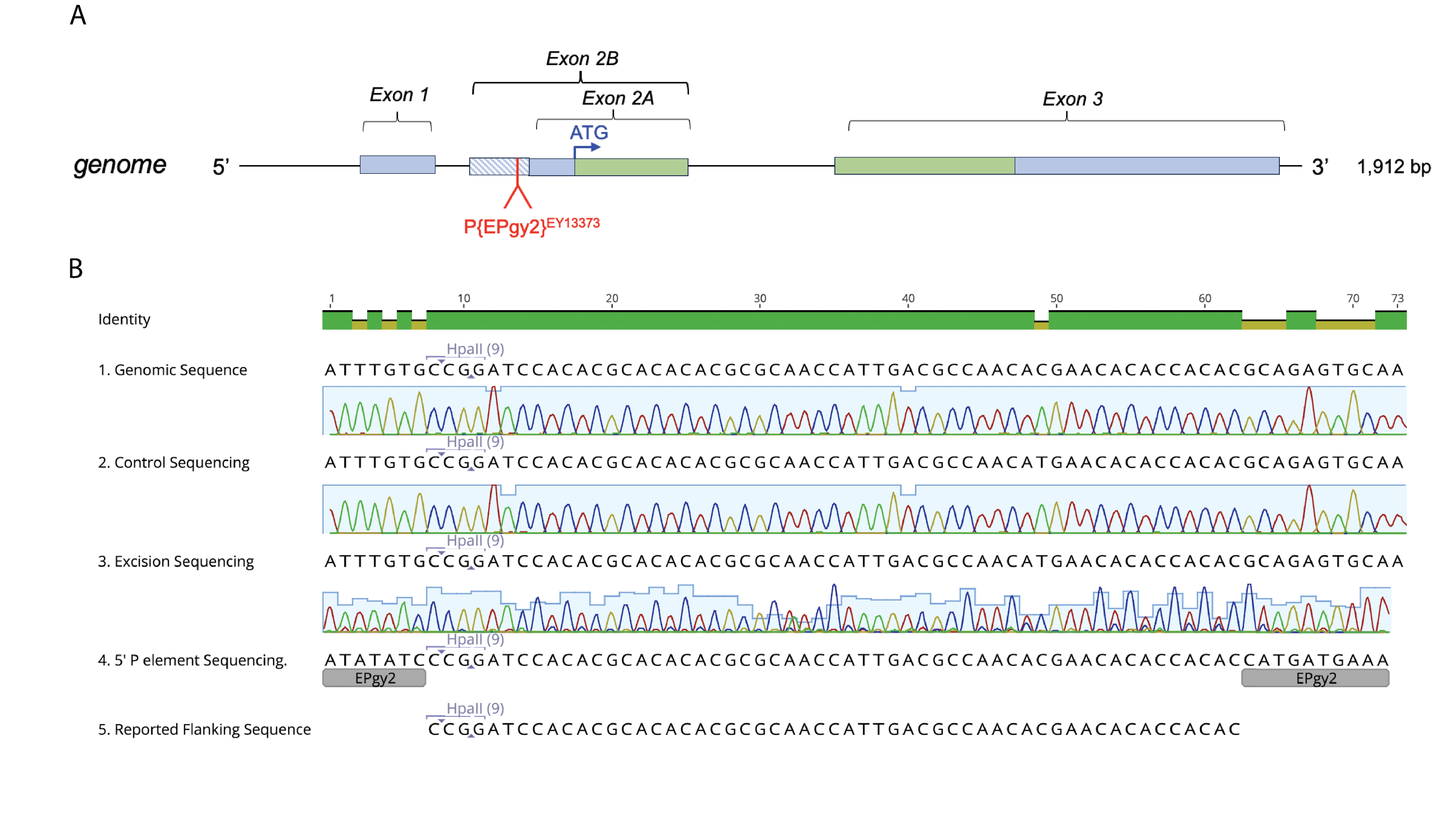

Supplement: S3 Fig — (A) Schematic of the PhLP3 gene region. The location of the P element insertion P{EPgy2}EY13373 is indicated 78 bp upstream of the translation start site in the 5’ UTR. (B) Sequence around the insertion site of P element. The following sequences and sequence data are represented: genomic sequence from FlyBase (FBgn0037843), sequencing result of the control strain (w1118), sequencing result of the PhLP3ΔP/ΔP excision strain, inverse PCR (iPCR) sequencing result of the P element insertion strain (PhLP3EY13373), and the previously reported flanking sequence for the P element. Sites, where HpaII would cut the sequence, are indicated, as this enzyme was used for iPCR. The sequenced regions of the P element insertion strain that do not align with the consensus sequence corresponding to the sequence from the P element, P{EPgy2}, not the PhLP3 gene. (TIF) [file pone.0306676.s003.tif]

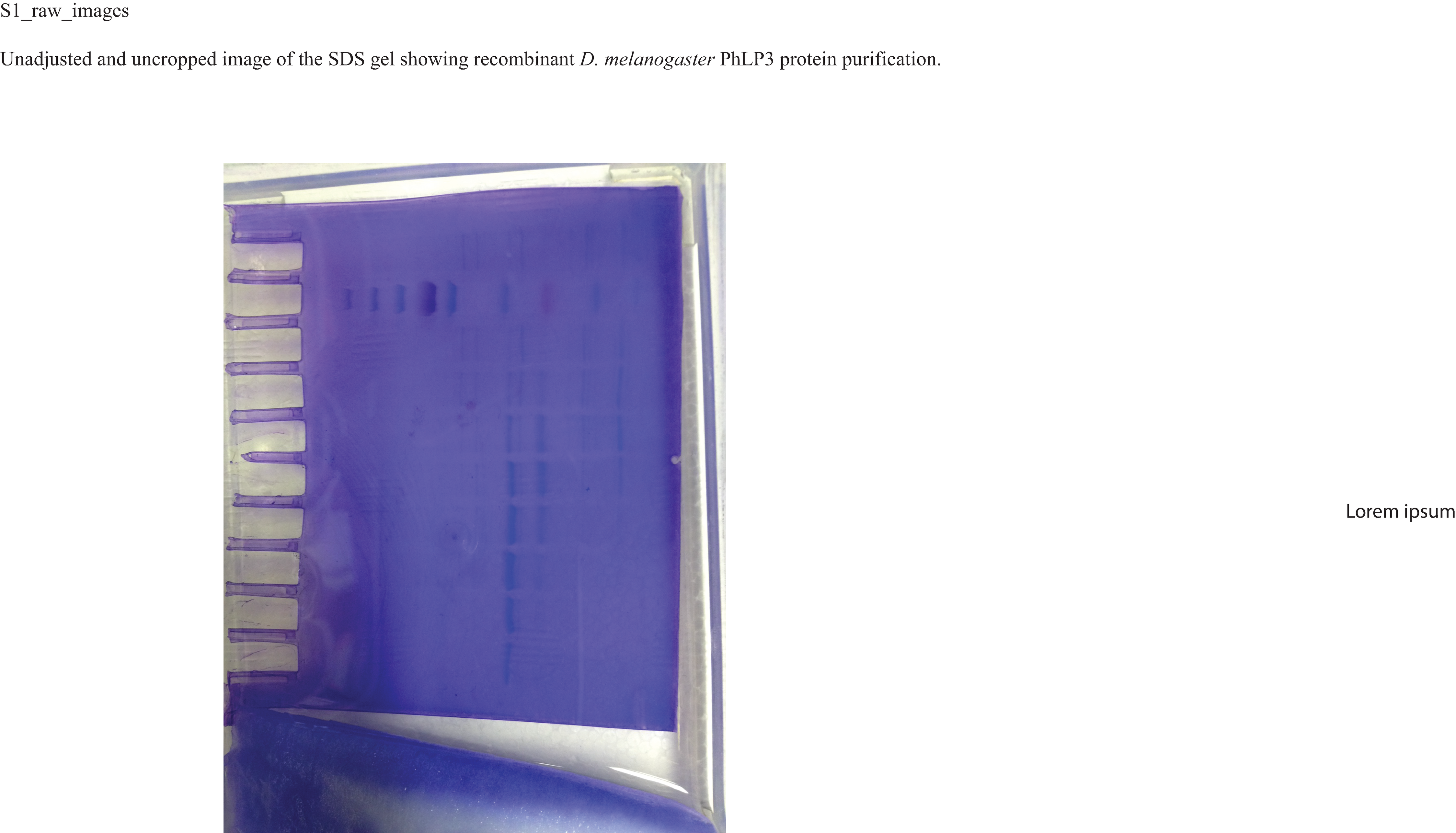

Supplement: S1 Raw image — (TIF) [file pone.0306676.s005.tif]
