# Supplementary material for: The evolutionarily conserved PhLP3 is essential for sperm development in Drosophila melanogaster
Source: PLoS One. 2024 Oct 31;19(10):e0306676. doi: 10.1371/journal.pone.0306676 (PMC11527243; doi:10.1371/journal.pone.0306676)
Supplement: S1 Table — (DOCX) [file pone.0306676.s004.docx]

| Gene | Forward | Reverse |
| --- | --- | --- |
| *RT-qPCR* |  |  |
| GAPDH_1 | AGTGTGACCTACGCAGAAAG | CGTTCAGTTTCAGTTCGCTATTAC |
| RpL32 | GGATTCAAGAAGTTCCTGGTGCAC | CGACAATCTCCTTGCGCTTCTTG |
| PhLP-3_1 | GCAAGGTGAATGCCGAAAAGACTCC | CGAAGTCGTCGCAGTTGCCC |
| PhLP-3_A | CTTCATTGTCGGATTCACCGATCTGG | CATCAGATCGCCCTTGTAGTCAATGG |
|  |  |  |
| *Alternative Splicing* |  |  |
| PhLP_3A_UTR | CAAACGGTCACACGAATCCATACG | CTGCTCCTCTGCGTGTCTTG |
| PhLP_3B_UTR | CGCACACACGCGCAACCATTG | GTTGTCCTCTTCCAGTTGCTGC |
|  |  |  |
| *Cloning* |  |  |
| PhLP-3 | GCCAACATCCTGGAGAACCAAC | TTAGTCATCCAGATCTATATCGGAGTC |
|  |  |  |
| *Sequencing* |  |  |
| PhLP3_Gene | GCCGATAAGGAAGCGCACC | GCTCGGTGTAGGTGCCATGA |
| Plac1 | CACCCAAGGCTCTGCTCCCACAAT |  |
| Pwht1 | GTAACGCTAATCACTCCGAACAGGTCACA |  |
| 5.SUP.seq1 | TCCAGTCACAGCTTTGCAGC |  |
