# Supplementary material for: The evolutionarily conserved PhLP3 is essential for sperm development in Drosophila melanogaster
Source: PLoS One. 2024 Oct 31;19(10):e0306676. doi: 10.1371/journal.pone.0306676 (PMC11527243; doi:10.1371/journal.pone.0306676)
Supplement: Attachment — Submitted filename: Response to Reviewers_FINAL.pdf [file pone.0306676.s006.pdf]

8/31/2024

Response to Reviewers

Dear Editor and Reviewers,

We sincerely thank you for your time, consideration, and careful review of our manuscript. According to your suggestions, the manuscript has been revised where applicable. Please find our responses to all the issues raised in blue below. Our answers are organized by reviewer number.

Reviewer #1

-Give units for the distance measure included at the top of the phylogenetic tree in Fig. 1B.

We labeled the scale of the phylogenetic tree and added an explanation to the legend: *The scale represents the "length" of the branches, which indicates the evolutionary distance between the sequences in units of amino acid substitutions per site. Longer branches represent larger numbers of genetic changes.*

-Specify in the caption whether the structure modeled in Fig. 1C is the *Drosophila* PhLP3 or another ortholog.

We have removed the 3D model of *Drosophila* PhLP3 from Fig 1, as it does not add to the introduction. We have generated a new Fig. 4 focusing on *Drosophila* PhLP3 structure prediction and modeling (see explanation in the next paragraph).

-If you haven't already, consider comparing the SWISS-MODEL structure to the AlphaFold prediction (https://alphafold.ebi.ac.uk/entry/Q9VGV8) to determine which is the better one to include in the figure. To my non-expert eye, they appear fairly similar, though the AlphaFold prediction shows a longer second alpha helix at the N-terminus (in line with the discussion section description, line 549). If you feel SWISS-MODEL is better, then no need to change. If you think the AlphaFold prediction is more reliable, then you could do the same for the structures in Fig. S1.

Thank you for your suggestion. We have considered the AlphaFold prediction, which coincidentally is the first template suggestion in SWISS-MODEL. We agree that the AlphaFold prediction shows the entire structure of *Drosophila* PhLP3, most notably the N-terminal helices and the thioredoxin domain, with a high confidence index. We generated a new Figure 4 that compares the AlphaFold and SWISS-MODEL structures of *Drosophila* PhLP3. We included the SWISS-MODEL shown in Fig 4B, as it is based on the experimentally determined structure of human PhLP2A. Notably, the template file with the PDB code 7NVM contains the CryoEM structure of the entire chaperonin TRiC/CCT complexed with PhLP2A and actin inside its luminal catalytic cavity. We hypothesize that *Drosophila* PhLP3 interactions with Drosophila TRiC/CCT are similar. Even though the PhLP2A structure is truncated, and consequently our *Drosophila* PhLP3 model too, it represents the structure under functional, cellular conditions.

-If any amino acids are missing from the structure, you could clarify this in the caption (e.g., it looks like positions 1-18 and 185-216 are not represented?).

In the new Fig. 4A (AlphaFold prediction), we highlighted the N-terminal and C-terminal sections missing in Fig. 4B (SWISS-MODEL). We explained the missing section in the main text (lines 179 – 181): *Notably, the first 18 N-terminal AA and the last 25 AA at the C-terminus were not modeled, as they were unresolved in the hPhLP2A crystal structure within the chaperonin complex.*

Line 114: The data in Fig. S2 do not seem to distinguish between "germline" and somatic cells of the testis, so consider re-wording this sentence (or, clarify that germline-specific expression is based on scRNA-seq data and/or your later data in Fig. 6, rather than the graphs in the supplemental figure?).

Line 114 (now line 115) We have added panel C to Fig. S2, which includes the scRNA-Seq data that illustrates PhLP3 expression in the germline from Li et al., 2022.

Figure 2:
-Add to the caption that "dc" indicates the dense complex in Fig. 2B.
Line 151: no italics for "mutants"

Figure 2: We have edited the Figure 2B legend, adding "dc: dense complex."

Line 151: no italics for "mutants"

Line 151 (now line 153) "mutants" is no longer italicized.

Figure 3:
-If possible, remove Microsoft's red-squiggly line under Trx.

Figure 3: The squiggly lines under Trx have been removed.

Figure 4:
-Fig. 1 also presents a PhLP3 structure and an alignment, so aspects of these figures are redundant with each other. Since there doesn't appear to be anything anomalous in the alignments or structures that is noteworthy for the manuscript (i.e., the structures align well, the cysteine is conserved, etc.), can these figures be condensed into one, with any other information being moved to supplemental material?

Figure 4 (new) Thank you. Following your suggestion, we have removed the SWISS-MODEL from Fig. 1 and generated a new Fig. 4 that shows the AlphaFold prediction as well as the SWISS-MODEL of *D. melanogaster* PhLP3. Fig. 1 A and B relate to the introduction while the modeling was performed in this study and is, therefore, part of the results section. We moved all structures to Figure 4.

-In the caption describing Fig. 4A, clarify which structure is shown in part with the green curves.

Figure 4A (now 4B): We added the following explanation to the legend of our new Fig 4B: "*The green portions of the PhLP3 structure represent unstructured segments, including loops and turns*."

-Line 190: unnecessary underlining of ref. [16].

Line 190 (now 197): ref [16] is now properly formatted and listed as ref [32] and is no longer underlined.

Line 200: it may be helpful to give a brief (one line) description of "the thioredoxin system" here, since this system might be unfamiliar to some readers, or simply add "(describe in the next paragraph)", where it's clarified.

Line 200 (now line 210): We have included the following sentence at the beginning of that paragraph. "*The cytosolic thioredoxin system is central in distributing electrons to target proteins in various antioxidant and regulatory pathways*."

Line 202: Do you mean Cys95 in D. melanogaster, rather than Cys93? (Also, be consistent with how you write the amino acids; cysteine is abbreviated as C in line 195, Cys here.)

Line 202 (now line 215): We corrected CYS93 to CYS95 and used this format throughout the manuscript.

Figure 5:
-It may be helpful to show the chemical reaction in Fig. 5B with the same protein names as you use in the text. For example, in line 215, you write about D. melanogaster "Trx1", but it's just "Trx" in Fig. 5B. Overall, these look like great data!

Figure 5: As CG4193 is annotated as DmTrx-1 in FlyBase, we therefore adjusted Trx and Trx1 annotations to Trx-1 or DmTrx-1 throughout the manuscript where appropriate and for consistency, including Fig. 5B.

Figure 6:
-The first line of the figure caption (line 243) states that panels "(A-B)" show in situ data, but it looks like only panels A and C show this, while panel B shows qPCR data. Please clarify.

Figure 6: We updated the labeling within the Figure 6 legend (now line 256) and the figure description in the text to match. A new panel was added to show the germline staining with anti-Vasa alone compared to the PhLP3 *in situ* to better demonstrate PhLP3 expression in the germline.

Figure 8:
-To support the text on lines 322-323, is it possible to show images with only the phalloidin channel here, to illustrate the presence of ICs for controls and the absence of them for the mutants? (If not, that is okay; this is a small point.)

Figure 8: Text lines 322-323 (now 344-345): We added new sets of panels showing only the phalloidin channel.

Figure 9:
-Is it possible to add to the caption whether these images represent zoomed in portions of whole-mount (intact) testes (which seems to be what the Methods section implies on line 450), or whether they come from shreds to release spermatid bundles?

Figure 9: We modified the caption to specify that these images were taken from whole mount testes.

It is a bit surprising that the P-element excision line (with 33% of WT levels of PhLP3 transcripts) is fertile, while the RNAi line (with 23% of WT levels of PhLP3 transcripts) is near-sterile. Either the authors have serendipitously narrowed down the exact threshold level of PhLP3 transcripts required for functional spermatogenesis, or there are other biological or technical explanations. (Example biological: there could be post-transcriptional regulation that acts differently between the delta P line and the RNAi progeny or some strain background differences with the RNAi line. Example technical: there could be variation in the RT-PCR measurements. The bam-gal4 control line measurement in Fig. 10 has large error bars, for instance, while error bars appear to be missing for the +/+ genotype in Fig. 6.) This doesn't require extensive comment, but if the authors had thoughts about it, they could briefly include them.

The reviewer describes the exciting observation that flies with just 33% of PhLP3 expression (P element excision line) relative to controls produce sperm and are fertile. In comparison, those with 23% of PhLP3 expression (RNAi line) relative to controls are sterile and fail to produce mature sperm. As suggested by the reviewer, it is possible that our results indicate a critical threshold for PhLP3 expression required for protein function during sperm development. It is notable that the controls for each of these experiments are in different genetic backgrounds, and therefore, the levels across controls may not be the same. We have added some discussion about this observation and these possibilities to the final paragraph of the discussion section.

Figure 10:
-Optional: if possible, it would be useful to show the scattered actin cones in the RNAi flies at higher magnification, but in my view, it is not necessary to re-do this experiment if you don't already have the data available.

Fig. 10: We added 20X pictures (panels H and N) to illustrate the RNAi phenotype and the actin cones.

Line 419: "or control" doesn't need to be in italics

Line 419 (now line 442): Removed italics of "or control."

Line 474-475: it's not clear why Trx1 primers were used, since the manuscript doesn't report on Trx1 purification. If the authors needed to produce and purify Trx1 for the biochemical assay, this can be briefly added to the manuscript, or they can simply state that Trx1 was produced as previously described in ref. 4.

Line 474-475 (now 497-498): We removed the DmTrx-1 primers from Table S1 and added the suggested statement in the Enzyme assays section (line 512-513) "*D. melanogaster thioredoxin-1 (DmTrx-1, CG4193, [37]) was produced as described previously [4,35]*". We added another reference [35] (Kanzok et al. 2001); we corrected the naming of Trx to Trx-1 for consistency, as requested earlier.

Given the high expression of CG4511 in ovaries, did the authors observe whether homozygous P-element insertion females were sterile?

We appreciate the reviewer's question about the fertility of homozygous P-element insertion females. We have observed that these females are sterile, and we are currently studying the effect of PhLP3 mutation on oogenesis. We plan to publish this work soon when we have more information on the role of PhLP3 in females. Therefore, we chose not to include the data here.

Lines 568-569: Related to the point above: This part of the discussion seems to raise the possibility that PhLP3 could be a redox target of Dhd in the ovaries. There doesn't yet appear to be any evidence that PhLP3 is part of mature sperm (for example, while not definitive evidence for absence, CG4511 was not detected amongst the 3,000+ sperm proteome proteins; Garlovsky et al. 2022, PMID: 35985624), so it isn't clear that testis-expressed PhLP3 would have the opportunity to interact with Dhd. Alternatively, since the data in Fig. S2 show high PhLP3 expression in ovaries as well as testes, it could be possible that ovary-expressed PhLP3 is a target of Dhd. Thus, consider rephrasing here.

Lines 568-569 (now lines 594-595): The reviewer mentions the lack of detection of CG4511 among the sperm proteome proteins in Garlovsky et al., 2022. This study utilizes sperm isolated from the seminal vesicle for these proteome studies. Our data suggests that CG4511/PhLP3 functions prior to sperm reaching maturity and being transferred to the seminal vesicle. It is possible that PhLP3 may no longer be expressed and required in mature sperm. Interestingly, Dhd is highly expressed in male germline cells of the testis, based on scRNA-seq data. Considering the ubiquitous presence of Dhd, this supports the hypothesis of an interaction between PhLP3 and Dhd.

Line 571-572: Clarify this sentence a bit. What aspect of the kinetics data presented here (and, compared to what other data) suggests that PhLP3 is unlikely to be an effective antioxidant scavenger?

Lines 571-572 (now lines 597-606): We thank the reviewer for pointing this out. The original sentence suggests that we tested the antioxidant activity of *Drosophila* PhLP3, which we did not. We corrected the statement as follows: *The thioredoxin system is a central electron distribution system. Some of its target proteins are thioredoxin-like proteins with strong antioxidant activity [56]. We previously demonstrated that P. berghei PhLP3 and its homolog human TXNDC9 exhibit antioxidant activity that is orders of magnitude weaker than that of antioxidant effectors, such as thioredoxin-dependent peroxiredoxin (TPx1) [4]. We concluded that PhLP3 is unlikely to act as an antioxidant scavenger in the cell. In the fruit fly effective antioxidant systems, such as catalase and superoxide dismutase, cooperate in the fly to protect from reactive oxygen species [57]. The hypothesis for a different function of PhLP3 is corroborated by the recent structural insights into the interactions of PhLP3 and PhLP2A with the CCT complex and its potential role in the folding of actin and tubulin [6,17].*

Line 638: remove the extra) after the parentheses

Line 638 (Now line 668): extra parenthesis removed

Line 641: "…described by Fabrizio have … [68]."

Line 641 (now line 671): removed "1998", as this detail is evident with the citation [68]

Reviewer#2:

i. In line 349-351, the authors denote that the p-element excision used in the article only restores expression to the level of 33%, but restores fertility in the animals. However, knockdown by RNAi (by 77%, as per line 356-357) is sufficient to render the animals infertile. Based on the p-element data, I would expect the knockdown to not render the animals sterile based on expression. I believe that further explanation is required beyond the expression levels to be confident in the validity of this data.

Line 349-351 (now 371-375): We have provided additional hypotheses in the discussion (lines 695-703) that could explain the restoration of fertility in the excision line with only 33% restoration of gene transcription while in RNAi experiments, knock-down to 23% PhLP3 expression relative to controls results in sterility. It is possible that our data reveal a critical threshold for PhLP3 expression levels that are required for sperm development. We also considered that these experiments were performed in different genetic backgrounds, which could contribute to the relative expression levels. As we are assaying mRNA levels in these experiments and do not know how stable the protein is, it is also possible that protein levels are significantly different across these experiments. This was added to the final paragraph of the discussion (lines 695-703).

ii. Only one RNAi line is used in the paper. I would much prefer to see at least two different lines, especially given the potential issue raised prior.

We used the only available RNAi strain that is not predicted to have off-target effects. Two additional RNAi lines are available: strain v34861 from the *Vienna Drosophila Resource Center*, Austria, and strain HMJ21142 from the *National Institute of Genetics*, Japan. Both lines are predicted to have off-target effects.

Reviewer#3:

Line 114 "high expression in the testis, specifically in germline cells (S2 Fig)"I do not see any evidence for expression in germline cells in the figure. Is there any more data?

Line 114 (now line 115): We added a panel to Fig. S2 (Fig. S2 C) that illustrates the scRNA-seq data highlighting the germline-specific expression of PhLP3/CG4511. The red dots in the left image indicate the expression in germline cells, which are indicated in green in the right image.

Line 170 " an N-terminal helix domain, the central Trx-domain, and the unstructured N-terminal tail" "unstructured N-terminal tail" is right or unstructured C-terminal tail? This is because "mostly unstructured C-terminal tail" appears online 543.

Line 170 (now line 173): Thank you for spotting this. We have corrected the "unstructured N-terminal tail" to "unstructured C-terminal tail."

Line 239 "gene is expressed in germ cells" It is difficult to see the exact location of expression in the figure. It may be recommended to add an enlarged view of A.

Line 239 (now line 252): Thank you for this suggestion. We added a set of panels To Fig.6 (Fig. 6 B and E) showing the anti-Vasa stain alone, which labels the germline. It highlights the presence/absence of PhLP3 in the germ cells (Fig. 6 C and F).

Figure 7
Nonparametric Welch's t-test is recommended, particularly between the control and mutant as in Figure 10.

Figure 7 and lines 280-281: As recommended, we utilized Welch's t-test for the data in Fig.7 to examine the significance of the data in a pairwise fashion, as we did in Figure 10. This slightly changed the p values, but the statistical significance was not affected.

Fig. 8 and line 322
It is difficult to see phalloidin staining (actin cones) in A and also to find sperm in seminal vesicles. Is it possible to add more focused and enlarged view? In addition, it may also be better to combine Figures 8 and 9 in order to understand the phenotypes.

Figure 8 and line 322 (now line 344): As recommended, we added a set of panels for each genotype showing phalloidin staining alone (Fig. 8 C, H, M) to illustrate the actin cones better. We added panels showing an enlarged view of the seminal vesicles (Fig 8 E, J, O), so sperm or lack thereof are more accessible to visualize. We also expanded Figure 9 with panels showing phalloidin staining alone (Fig. 9 B and E) and panels merged with DAPI staining (Fig. 9 C and F).

Figure 9
It takes time to understand which are open and solid arrows; alternative symbols are better.

Figure 9: Thank you for the suggestion. We now use the following symbols to label the three cell types: *Arrowheads* label canoe stage nuclei, *dashed arrows* label leaf stage nuclei, and *solid arrows* label needle-like nuclei. We have specified the new arrow types in lines 334–335.

Figure 10
"White arrows indicate clusters of elongating spermatid nuclei" This is phalloidin staining; the revision is needed. There are no "arrows" in C. I cannot see evidence for "there are few if any DAPI clusters" in D or cannot understand where I should see. It is also difficult to see exactly "deformed, scattered actin cones." Because this is very important point of this paper, the authors should be strongly recommended to prepare double staining and enlarged views for this.

Figure 10: Per your suggestion, we have significantly updated Fig. 10 by adding panels that show single and double labeling at a higher magnification, which better illustrates the phenotype. We have expanded and corrected the description of each panel and specified what arrows are pointing out within each panel in the Figure legend (see lines 406-421 for the corrected figure legend).

Line 460 "manufacturer's instructions" Which is the manufacturer?

Line 460 (now line 482): We added the name of the manufacturer: Molecular Research Center (MRC).

Line 549 "Stirling et al." Reference number [6] should be just after the author's name.

Line 549 (now line 574): We moved the citation [6] behind the author's name.

Line 613 "Others" Reference or references should be provided here.

Line 613 (now line 644): Thank you for the suggestion. We added the two references for this statement:

Fuller MT, Caulton JH, Hutchens JA, Kaufman TC, Raff EC. *Genetic analysis of microtubule structure: a beta-tubulin mutation causes the formation of aberrant microtubules in vivo and in vitro*. J Cell Biol. 1987;104: 385–394. doi:10.1083/jcb.104.3.385
Li P, Messina G, Lehner CF. *Nuclear elongation during spermiogenesis depends on physical linkage of nuclear pore complexes to bundled microtubules by Drosophila Mst27D*. PLOS Genet. 2023;19: e1010837. doi:10.1371/journal.pgen.1010837

Reviewer#4

Major comments

1. It needs to be clear the reason of male sterile is due to the early release of mature sperm in the experimental group leads to infertility or the absence of spermatid extension stage abnormalities leads to abnormal individualization.

This is an interesting argument. However, the scattered sperm observed in PhLP3 mutants do not appear to be mature sperm, as signified by their abnormal nuclear shape, the absence of actin cones throughout the testis, and a failure to transfer to the seminal vesicle. We hypothesize that the scattering of immature sperm within a syncytium and the absence of mature sperm is the cause of the infertility phenotype. Our data suggests defects in nuclear elongation and individualization before sperm release, leading to an arrest in sperm development. We hypothesize that the failure to produce mature sperm that can be transferred to the seminal vesicle is the cause of the infertility phenotype. We are currently investigating this and other theories and look forward to sharing our findings soon.

2. PhPL3 is a chaperone protein involved in protein folding as well as a role as an enzyme. Whether the protein degradation pathways were activated in the absence of PhPL3, such as ubiquitination proteasomal degradation pathway, lysosomal pathway, caspase. It is preferable do detect some gene expression related to these pathway.

Thank you for your suggestions. Here, we describe the initial characterization of *Drosophila* PhLP3 and a surprising phenotype. Your suggestions are appreciated and will help us plan important next steps in investigating and determining the function(s) of PhLP3 as a chaperone, its potential effect on protein quality, and its role in the *Drosophila* germline. We look forward to exploring these roles in the future.

3. It is preferable do detect some gene expression related to spermatogenesis in the absence of PhPL3.

Thank you for your suggestion. As no markers are yet available to distinguish between the different spermatid stages, DAPI staining was the best option for this initial phenotype analysis and characterization. We are delving deeper into the *PhLP3* mutant phenotype to explore other aspects of sperm maturation that may be affected, including chromatin condensation, the histone to protamine shift, and potential changes in gene expression as a consequence of PhLP3 mutation. Given the failure to produce mature sperm, we expect significant changes in gene expression due to the loss of different cell types in the testis.

4. In the result session, authors should explain the reason of using bam-gal4.

New line 379: We added a citation for the GAL4/UAS system in line 359 (Brand and Perrimon 1993). In lines 384-385, we clarified that bam-Gal4 was specifically used to initiate expression of the UAS-Phlp3-RNAi and when it drives target gene expression. We added the citation related to the testis expression of bam-Gal4 (White-Cooper 2012).

5. It is perferable to mark "seminal vesicle" in figure 8 and 10, thus easy for readers.

White square brackets were added to Fig.8 (D, I, and N) and Fig. 10 (G and M) to indicate the seminal vesicles.

6. "***" shouled be marked in figure 8A.

*** relates to the original Figure 8D (now 8P) and the legend states*: **** indicates p<0.0001 and *** indicates p<0.001 based on ordinary one-way ANOVA.*
